# Glycyrrhizin, inhibitor of high mobility group box-1, attenuates monocrotaline-induced pulmonary hypertension and vascular remodeling in rats

**DOI:** 10.1186/s12931-014-0148-4

**Published:** 2014-11-25

**Authors:** Pil-Sung Yang, Dae-Hoon Kim, Yong Joon Lee, Sang-Eun Lee, Won Jun Kang, Hyuk-Jae Chang, Jeon-Soo Shin

**Affiliations:** 1 Division of Cardiology, Severance Cardiovascular Hospital, Yonsei University Health System, 50 Yonsei-ro Seodaemun-gu, Seoul, 120-752 Republic of Korea; 2 Departments of Microbiology, Yonsei University College of Medicine, 50 Yonsei-ro Seodaemun-gu, Seoul, 120-752 Republic of Korea; 3 Departments of Nuclear Medicine, Yonsei University College of Medicine, 50 Yonsei-ro Seodaemun-gu, Seoul, 120-752 Republic of Korea; 4 Severance Biomedical Science Institute, Yonsei University Health System, 50 Yonsei-ro Seodaemun-gu, Seoul, 120-752 Republic of Korea

**Keywords:** High mobility group box-1 (HMGB1), Pulmonary hypertension, Pulmonary vascular remodeling, Glycyrrhizin, Inflammation

## Abstract

**Background:**

High mobility group box-1 (HMGB1), a proinflammatory cytokine, plays a pivotal role in tissue remodeling and angiogenesis, both of which are crucial for the pathogenesis of pulmonary arterial hypertension. In this study, we explored the relationship between HMGB1 and pulmonary hypertension and whether glycyrrhizin, an inhibitor of HMGB1, attenuates disease progression in an animal model of pulmonary hypertension induced by monocrotaline sodium (MCT).

**Methods:**

After inducing pulmonary hypertension through a single subcutaneous injection of MCT (60 mg/kg) to Sprague–Dawley rats, we administered daily intraperitoneal injections of either glycyrrhizin (GLY, 50 mg/kg), an inhibitor of HMGB1, or saline (control) for either 4 or 6 weeks.

**Results:**

Expression levels of HMGB1 in serum increased from the second week after MCT injection and remained elevated throughout the experiment periods. Lung tissue levels of HMGB1 assessed by immunohistochemical staining at 4 weeks after MCT injection also increased. Chronic inhibition of HMGB1 by GLY treatment reduced the MCT-induced increase in right ventricular (RV) systolic pressure, RV hypertrophy (ratio of RV to [left ventricle + septum]), and pulmonary inflammation. MCT-induced muscularization of the pulmonary artery was also attenuated in the GLY-treated group. As assessed 6 weeks after MCT injection, the GLY-treated group exhibited increased survival (90% [18 of 20]) when compared with the control group (60% [12 of 20]; p =0.0027).

**Conclusions:**

Glycyrrhizin, an inhibitor of HMGB1, attenuates pulmonary hypertension progression and pulmonary vascular remodeling in the MCT-induced pulmonary hypertension rat model. Further studies are needed to confirm the potential of HMGB1 as a novel therapeutic target for pulmonary hypertension.

## Background

Pulmonary arterial hypertension is a fatal disease characterized by elevated pulmonary vascular resistance that ultimately leads to right ventricular (RV) failure and death [1]. Despite current therapeutic approaches for improving symptoms, exercise capacity, and hemodynamic variables, they still have a limited effect on vascular remodeling and do not confer a mortality benefit [2,3].

Like many other vasculopathies, pulmonary hypertension is characterized by severe angioproliferative vascular remodeling. Inflammation is considered to play a central role in disease progression in various types of pulmonary hypertension [4-8]. Serum levels of multiple chemokines and cytokines related to inflammatory processes are elevated, and inflammatory cells such as macrophages, T and B lymphocytes, and dendritic cells infiltrate vascular lesions in pulmonary hypertension patients.

High mobility group box-1 (HMGB1) was initially identified as a nuclear non-histone DNA-binding protein, which stabilizes nucleosome structure and regulates transcription [9,10]. HMGB1 localizes to the nucleus under normal conditions and is released to the extracellular milieu in response to inflammatory stimuli. HMGB1 mediates various systemic inflammations such as sepsis, arthritis, and autoimmune diseases [11-13] by binding to cellular receptors such as the receptor for advanced glycation end products (RAGE), toll-like receptor (TLR)-2 and TLR-4 [14,15]. HMGB1 is also involved in tissue remodeling and angiogenic activities; it promotes migration and proliferation of smooth muscle cells [16,17] and mediates the activation and migration of endothelial cells [18,19].

Considering the pathophysiology of pulmonary hypertension and the role of HMGB1 in inflammatory processes and tissue remodeling, there is reasonable probability that HMGB1 may be associated with the pathogenesis of pulmonary hypertension, an angioproliferative vasculopathy. A recent study also reported that serum HMGB1 levels were higher in idiopathic pulmonary hypertension patients than in healthy controls, and HMGB1 may contribute to the pathogenesis of experimental pulmonary hypertension induced by chronic hypoxia [20].

In this study, we explored the relationship between HMGB1 and pulmonary hypertension, investigating whether HMGB1 inhibition by glycyrrhizin attenuates disease progression of pulmonary hypertension and in turn translates into mortality benefit, by using a well-established experimental pulmonary hypertension model induced by monocrotaline sodium (MCT).

## Methods

### Animal study design

**Figure 1:**
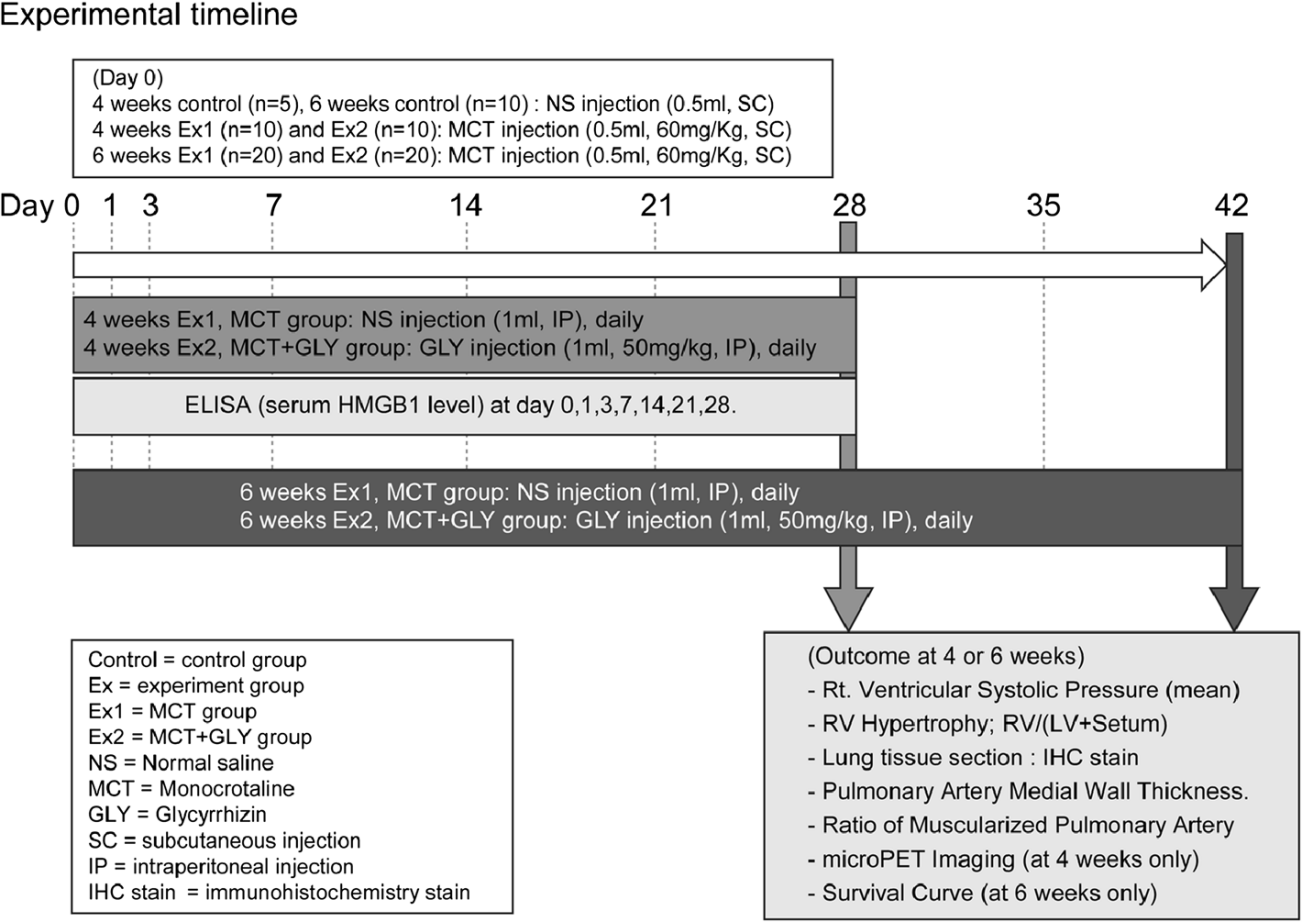
**Scheme of animal experimental timeline.** Pulmonary hypertension was induced by a single subcutaneous injection of MCT (60 mg/kg). The GLY (50 mg/k) was intraperitoneally treated once daily immediately after MCT injection for 4 weeks or 6 weeks in the MCT + GLY group, while normal saline was injected in the MCT group. Hemodynamic changes, RV hypertrophy, histological changes of lung, micro-PET, and survival rate were measured at the end of the animal experiment.

This animal study protocol was approved by Institutional Animal Care and Use Committee of Yonsei University Health System, in accordance with the Guide for the Care and Use of Laboratory Animals (National Research Council, USA). We performed animal experiments twice, for 4-week and 6-week periods, to evaluate both the established stage and the end stage of pulmonary hypertension. Male Sprague–Dawley rats (body weight 240–260 g) were randomly divided into 3 groups in each experiment: Group 1, initially given a single subcutaneous injection of normal saline and no treatment (control group; n = 5 [4 weeks] and 10 [6 weeks]); Group 2, initially given a single subcutaneous injection of MCT (0.5 ml, 60 mg/kg; c2401; Sigma-Aldrich) and once daily intraperitoneal treatment of normal saline (MCT group; n = 10 [4 weeks] and 20 [6 weeks]); and Group 3, initially given a single subcutaneous injection of MCT (0.5 ml, 60 mg/kg) and once daily intraperitoneal treatment of glycyrrhizin (GLY) (50 mg/kg; 50531; Sigma-Aldrich) (MCT + GLY group; n = 10 [4 weeks] and 20 [6 weeks]). After the above animal experiments, an additional experiment was performed to verify the effects of GLY alone without MCT on the pulmonary vascular system. An initial single subcutaneous injection of normal saline and once daily intraperitoneal treatment of GLY (50 mg/kg) for 4 weeks was performed in male Sprague–Dawley rats (GLY group; n = 6). The MCT was dissolved in 1.0 N HCl, and the pH was adjusted to 7.4 with 0.5 N NaOH. A dose of 50 mg/kg GLY was selected on the basis of previous animal studies using GLY [21,22]. A timeline of the animal experiments is illustrated in Figure 1.

### Measurement of the level of HMGB1 in the lung tissue and serum

Rat lung tissues were harvested 28 days after MCT injection and immunohistochemical staining with a mouse monoclonal anti-human HMGB1 antibody (ab18256; Abcam) was performed. Serum was separated 0, 1, 3, 7, 14, 21, and 28 days after MCT injection from the tail vein and stored in a -70^o^ C deep freezer. The HMGB1 level in the serum was analyzed by an ELISA kit (Shino-Test, Japan) in accordance with the manufacturer’s instructions.

### Measurement of RV pressure and hypertrophy, tissue preparation

Hemodynamic measurement and organ harvesting were performed at the end of the animal experiments, 4 weeks or 6 weeks post MCT or normal saline injection. To monitor hemodynamics, rats were anesthetized with Zoletil (50 mg/kg; Virbac) and Rompun (5 mg/kg; Bayer). Mechanical ventilation was applied with a tidal volume of 10 mL/kg and a respiratory rate of 60 breaths/min. The chest was opened, and a right heart catheter (PE-50 tube) was directly inserted into the RV through the cardiac apex for measurement of right ventricular systolic pressure (RVSP) with force transducers (Edwards Lifesciences Corporation). After measuring RV pressure, the lung vascular bed was flushed by injecting cold phosphate-buffered saline (PBS). The left lung was fixed in 10% neutral buffered formalin for histologic examination. The heart was separated from the thoracic cavity and the RV was dissected from the left ventricle (LV) and the septum (S). Each part of the heart was weighed to determine the degree of RV hypertrophy from the ratio of RV to [LV + S].

### Histologic examination of the lungs

Histologic examination of the lungs was performed as described previously [23]. Elastica-eosin staining was performed according to common histological procedures to identify the medial area between the internal elastic lamina and external elastic lamina. Sections were examined using a virtual microscopy system (dotSlide; Olympus, Japan). Forty muscular arteries with external diameters from 50 to 100 μm were analyzed for each animal. The percentage of medial wall thickness was calculated as 2 × media thickness/external diameter × 100. Medial wall thickness was measured along the shortest curvature of the vessel. Double immunohistochemical staining with mouse monoclonal anti-human alpha smooth muscle actin (α-SMA) antibody (diluted 1:400; M0851; DAKO) and rabbit polyclonal anti-human von Willebrand factor (vWF) antibody (diluted 1:1000; A0082; DAKO) was performed to assess the degree of muscularization of the pulmonary arteries. Sections were counterstained with hematoxylin and examined using the virtual microscopy system. Sixty small pulmonary vessels with external diameters from 10 to 50 μm were counted at × 400 magnification for each rat. Counted vessels were categorized as fully muscularized, partially muscularized, or nonmuscularized by observers blinded to treatment. Nonmuscularized arterioles were detected by endothelial anti-vWF staining.

### Measurement of the degree of pulmonary inflammation by micro-positron emission tomography (PET)

A micro-PET scanner (Concorde Microsystems Inc, USA) was used to detect and quantify lung inflammation. Micro-PET scanning was performed with an injection of 2–3 MBq of ^18^ F-FDG in the tail vein after 28 days of MCT injection (normal control group, n = 5; MCT group, n = 5; MCT + GLY group, n = 5). Subjects were fasted for at least 6 hours before scanning. Circular regions of interest were placed on the lung field, and the standardized uptake value (SUV), which represents pulmonary FDG uptake per unit volume, was calculated as [mean regions of interest count (cps/pixel) × body weight (kg)]/[injected dose (mCi) × calibration factor (cps/mCi)] using free software, Amide’s a Medical Image Data Examiner.

### Culture of human pulmonary artery endothelial cells (HPAEC) and endothelin 1 (ET-1) release assay

HPAEC (CC-2530; Lonza) were cultured in commercial HPAEC medium (EGM-2, Lonza) supplemented with penicillin (100 U/ml)–streptomycin (100 μg/ml), 10% fetal bovine serum (FBS) at 37^o^C in a humidified 5% CO_2_ incubator. All experiments were performed on endothelial cells within the fifth passage. HPAEC was seeded in 96-well plates (1×10^4^ cells/well) for 24 hours, and recombinant HMGB1 (R&D systems) was treated to HPAEC in various concentrations (10, 20, 30, and 40 ng/ml) for 24 hours. To inhibit the ET-1 release induced by HMGB1, anti-HMGB1 antibody (ab18256; Abcam) (0.2 μg/ml, 1 μg/ml) and anti-RAGE antibody (ab3611; Abcam) (0.2 μg/ml, 1 μg/ml) were co-treated with recombinant HMGB1 of 40 ng/ml. The culture supernatant was harvested, and ET-1 release was measured using a commercial human ET-1 ELISA kit (#900-020A; Enzo Life Sciences Inc.) in accordance with the manufacturer’s instructions.

### Culture of human pulmonary artery smooth muscle cells (HPASMC) and proliferation assay

HPASMCs were supplied from Lonza and cultured according to the supplier’s instructions. All experiments were executed on smooth muscle cells within the fifth passage. A proliferation assay was performed as described previously [24]. HPASMCs were seeded in 6-well plates (1×10^5^ cells/well) with commercial HPASMC basal medium (SmBM; Lonza) supplemented with 5% FBS and growth factors. The medium was replaced with serum-free SmBM 24 hours after seeding. After 16 hours, we divided HPASMCs into three groups. The first group was cultured with serum-free medium alone. The second group was cultured with the medium in the presence of 10% FBS. The third group was cultured with the medium in the presence of HMGB1 (30 ng/ml). HPASMCs were incubated for 3 days and counted at 1, 2, and 3 days. Trypan blue dye was used to evaluate the viability of cells. Experiments were performed two times in duplicate.

### Statistical analysis

All data are given as means ± SEM. Differences between the groups were assessed by analysis of variance and the Bonferroni post hoc test. The survival rate was presented as a Kaplan-Meier curve and compared by a log-rank test. A p-value less than 0.05 was considered significant.

## Results

### HMGB1 levels increased in the MCT-induced pulmonary hypertension rat model

To examine the relationship between HMGB1 and pulmonary hypertension, we investigated the patterns and levels of HMGB1 expression in lung tissues at 28 days and measured serum levels of HMGB1 at 0, 1, 3, 7, 14, 21, and 28 days after inducing pulmonary hypertension by MCT.

**Figure 2:**
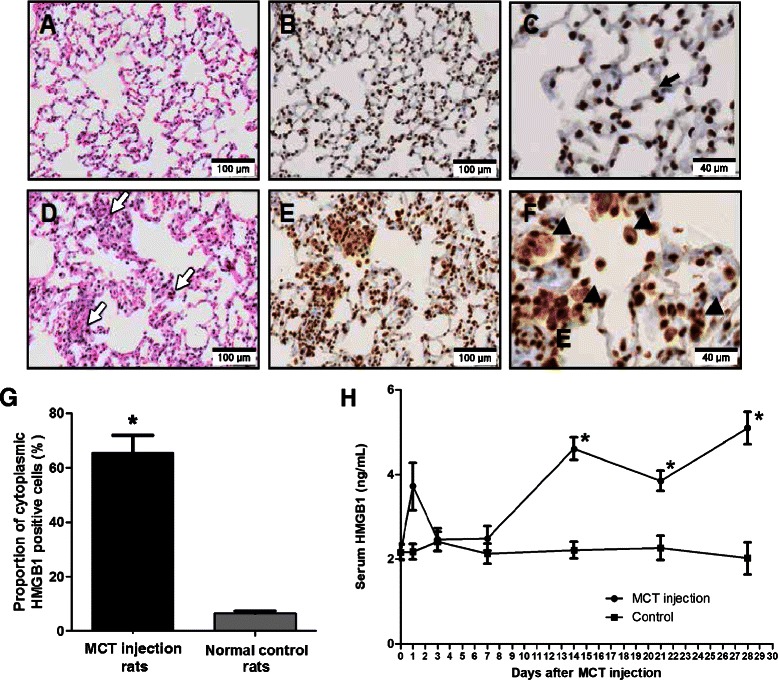
**Increased tissue and serum levels of HMGB1 in pulmonary hypertension rats induced by MCT. A–C**: Lung sections of normal control rats.** D–F**: Lung sections of MCT-induced pulmonary hypertension rats at day 28 after MCT injection. **A, D**: Hematoxylin and eosin staining. **B, C, E, F**: Immunohistochemical staining with anti-HMGB1 antibody (brown). **(A)** No inflammatory cell was observed in the lungs of normal control rats. **(B, C)** HMGB1 was mainly localized to the nucleus (black arrow). **(D)** A large number of inflammatory cells (white arrow) were recruited to the lungs of MCT-induced pulmonary hypertension rats. **(E, F)** HMGB1 translocated to the extranuclear area (triangle). **(G)** The proportion of cytoplasmic HMGB1 positive cells in lung tissue of MCT-induced pulmonary hypertension rats was greater than in that of normal control rats (*p <0.001). **(H)** Serum levels of HMGB1 in MCT-injected rats increased significantly 2 weeks after the MCT injection and remained elevated (*p <0.001 when compared to initial serum level of HMGB1). Data are presented as means ± SEM (n =10).

**Figure 3:**
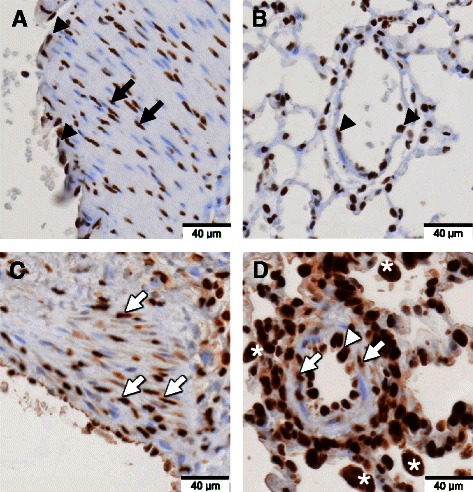
**Expression pattern of HMGB1 in the pulmonary vascular lesion.** Immunohistochemical staining with anti-HMGB1 antibody (brown color) and counterstaining with hematoxylin was performed on lung tissues of normal control rats and MCT-induced pulmonary hypertension rats at day 28 after MCT injection. **A, C**: Sections of large arteries. **B, D**: Sections of small vessels of lung tissue. **(A, B)** In normal control rats, HMGB1 was present only in the nuclei of smooth muscle cells (black arrow) and endothelial cells (black triangle). **(C, D)** However, in MCT-induced pulmonary hypertension rats, HMGB1 was present in both the nuclei and cytoplasm of smooth muscle cells (white arrow) and endothelial cells (white triangle). There were many inflammatory cells around the vessels with cytoplasmic HMGB1 translocation in MCT-induced pulmonary hypertension rats (white asterisk).

In lung tissues of the normal control rats, no inflammatory cells were observed as shown by hematoxylin and eosin staining (Figure 2A), and immunohistochemical staining with anti-HMGB1 antibody showed that HMGB1 was mainly localized to the nucleus (Figure 2B-C). However, in lung tissues of the MCT-induced pulmonary hypertension rats, many inflammatory cells were recruited to the lungs (Figure 2D), and HMGB1 was localized to both the nucleus and the cytoplasm (Figure 2E-F). Cytoplasmic translocation of cellular HMGB1 from the nucleus to the cytoplasm suggests subsequent release of HMGB1 into the extracellular milieu [25]. Cytoplasmic translocation of HMGB1 was also observed in vascular endothelial cells, smooth muscle cells, and recruited inflammatory cells in the MCT-induced pulmonary hypertension rats (Figure 3A-D). The proportions of cytoplasmic-HMGB1-positive cells in lung tissue was higher in the MCT-induced pulmonary hypertension rats (65.4% ±6.5%) than in the normal control rats (6.7% ±0.8%; p <0.001) (Figure 2G). Compared with the baseline, serum levels of HMGB1 measured by ELISA assays significantly increased 2–4 weeks after MCT injection (baseline: 2.2 ± 0.2 ng/ml; 2 weeks: 4.6 ± 0.3 ng/ml; 3 weeks: 3.9 ± 0.2 ng/ml; 4 weeks: 5.1 ± 0.4 ng/ml; all p <0.001), while control rats did not exhibit a significant increase of HMGB1 serum levels (Figure 2H).

### Inhibition of HMGB1 by GLY treatment reduced MCT-induced increases of RV systolic pressure, RV hypertrophy, medial wall thickness, and muscularization of pulmonary arteries

Based on our results linking HMGB1 and pulmonary hypertension, we tested the effects of GLY, an HMGB1 inhibitor, on hemodynamic features and vascular remodeling in MCT-induced pulmonary hypertension rats.

**Figure 4:**
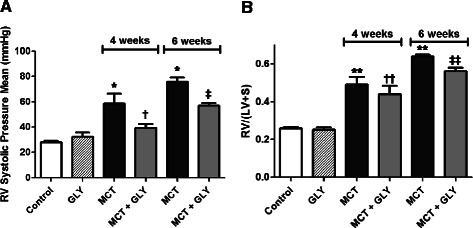
**Inhibition of HMGB1 by GLY treatment attenuated the MCT-induced increase of RV systolic pressure and RV hypertrophy. (A)** MCT-injected rats exhibited increased RVSP when compared with control rats (*p = 0.002). GLY treatment reduced the MCT-induced increase of RVSP (†p = 0.045 when compared to the MCT group treated for 4 weeks; ‡p = 0.002 when compared to the MCT group treated for 6 weeks). GLY treated alone without MCT yielded no significant difference in RVSP when compared with that of control rats (p = 0.307). **(B)** RV hypertrophy (RV/(LV + S)) increased significantly in the MCT group when compared with the control group (**p <0.001). GLY treatment reduced the MCT-induced increase of RV hypertrophy, although only rats given a 6-week GLY treatment course exhibited a statistically significant difference (††p = 0.418 when compared with the MCT group treated for 4 weeks; ‡‡p = 0.002 when compared with the MCT group treated for 6 weeks). GLY treated alone without MCT yielded no significant difference in RV hypertrophy when compared with that of controls (p =0.656). Data are presented as means ± SEM.

RVSP significantly increased in the MCT injection group at both 4 weeks (58.54 ± 7.67 mmHg) and 6 weeks (75.75 ± 3.21 mmHg) after MCT injection, compared with the control group (28.11 ± 0.85 mmHg; p = 0.002). Treatment of GLY reduced the MCT-induced increase of RVSP from 58.54 ± 7.67 mmHg to 39.17 ± 3.22 mmHg (4 weeks post-MCT injection; p = 0.045) and from 75.75 ± 3.20 mmHg to 56.75 ± 2.28 mmHg (6 weeks post-MCT injection; p = 0.002). In GLY given alone without MCT, there was no significant difference in RVSP when compared with the control group (p = 0.307). Therefore, the inhibition of HMGB1 by GLY treatment can attenuate hemodynamic changes in MCT-induced pulmonary hypertension rats (Figure 4A).

The ratio of RV to LV weight plus the S weight (RV/(LV + S)) expresses the degree of RV hypertrophy. Compared to the control group (0.2572 ± 0.0079), the RV/(LV + S) value increased significantly in the MCT groups at both 4 weeks (0.4898 ± 0.0404) and 6 weeks (0.6397 ± 0.0097) after the MCT injection (p <0.001). GLY treatment reduced the value of RV/(LV + S) from 0.4898 ± 0.0404 to 0.4403 ± 0.0437 at 4 weeks after MCT injection (p = 0.418) and from 0.6397 ± 0.0097 to 0.5596 ± 0.0204 at 6 weeks after MCT injection (p = 0.002) in the MCT + GLY group. In GLY given alone without MCT, there was no significant difference in the RV/(LV + S) value when compared with the control group (p = 0.656). These data demonstrate that RV hypertrophy developed in the MCT group and that this change could be attenuated through the inhibition of HMGB1 by GLY treatment (Figure 4B).

**Figure 5:**
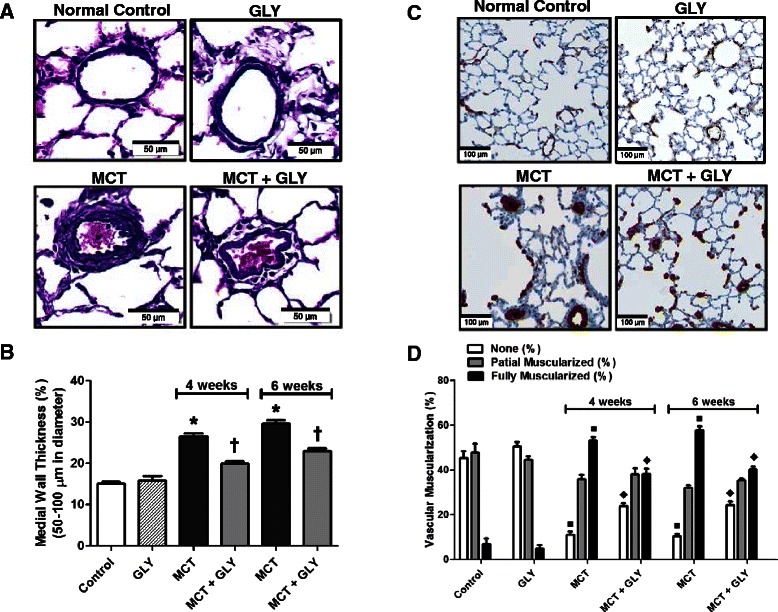
**MCT-induced increases in both medial wall thickness and muscularization of pulmonary arteries were attenuated through the HMGB1 inhibition by GLY treatment. (A, B)** Elastica-eosin staining showing that the medial area between the lamina elastica interna and externa (deep violet) increased in the MCT group when compared with the normal control group (*p <0.001). Treatment with GLY attenuated the medial change induced by MCT injection (†p <0.001). GLY treated alone without MCT yielded no significant difference in medial wall thickness when compared with that of control rats (p = 0.501). **(C, D)** The degree of muscularization of pulmonary arteries was observed via immunostaining of α-SMA (brown) and vWF (purple). In the MCT group, the proportion of muscularized pulmonary arteries increased when compared with the control group (■p <0.001). GLY treatment attenuated this MCT-induced muscularization of pulmonary arteries (♦p <0.001). GLY treated alone without MCT yielded no significant difference in pulmonary artery muscularization when compared with that of control rats (p = 0.262). Data are presented as means ± SEM.

The medial wall thickness (expressed as a percentage of total wall thickness) was analyzed in pulmonary arteries with external diameters between 50 to 100 μm. Elastica-eosin staining was performed to identify the medial area between the lamina elastica interna and externa. In the MCT group, the medial wall thickness significantly increased compared with the control group (control group: 14.98% ±0.56%; MCT group, 4 weeks: 26.51% ±0.68%; MCT group, 6 weeks: 29.59% ±0.83%; p <0.001). Upon treatment with GLY, the MCT-induced increase in medial wall thickness was attenuated from 26.51% ±0.68% to 19.93% ±0.62% at 4 weeks after MCT injection (p <0.001) and from 29.59% ±0.83% to 22.91% ±0.67% at 6 weeks after MCT injection (p <0.001) in the MCT + GLY group. GLY treated alone without MCT did not cause a significant difference in medial wall thickness when compared with the control group (p = 0.501) (Figure 5A-B).

The degree of muscularization of pulmonary arteries with external diameters between 10 to 50 μm was also analyzed. To this end, we performed double immunohistochemical staining of lung tissue with anti-α-SMA antibodies and anti-vWF antibodies. Staining with the former identified the smooth muscle layer of the vessel, whereas staining with the latter distinguished the endothelial layer of the vessel. In the MCT group, the amount of nonmuscularized pulmonary arteries decreased; however, the amount of fully muscularized pulmonary arteries increased compared to the control group (p <0.001). In contrast, upon GLY treatment, muscularization of pulmonary arteries in the MCT group significantly decreased at both 4 and 6 weeks in the MCT + GLY group (p <0.001). GLY treated alone without MCT displayed no significant difference in pulmonary artery muscularization when compared with the control group (p = 0.262) (Figure 5C-D).

### Inhibition of HMGB1 by GLY treatment improved the survival rate and reduced inflammation of the lung in the MCT-induced pulmonary hypertension rat model

**Figure 6:**
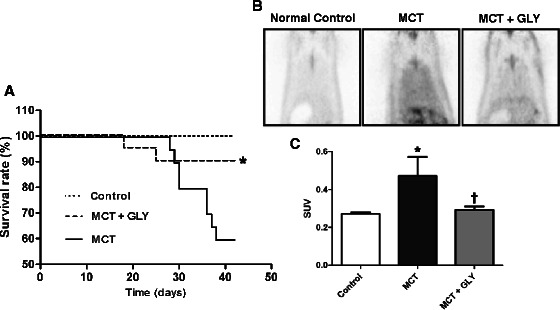
**Effect of GLY treatment on survival rate and pulmonary inflammation in the MCT-induced pulmonary hypertension rat. (A)** GLY was administered for 42 days, and rat survival was compared with the non-treated MCT group. The survival rate was 60% in the non-treated MCT group; in contrast, the survival rate was 90% in the MCT + GLY group (*p = 0.0027). **(B, C)** The degree of pulmonary inflammation was measured by micro-PET at 28 days post-MCT injection. The SUV of the lung field increased in the MCT group compared with the control group (*p = 0.009). GLY treatment reduced the SUV of the lung field when compared with that of the non-treated MCT group (†p = 0.018). Data are presented as means ± SEM.

Next, we observed whether inhibition of HMGB1 influences survival. For this purpose, MCT-injected rats were treated with GLY, and their survival rate was compared to that of rats in the MCT group. In the MCT group, the survival rate decreased to 60% (12 of 20 animals) at 42 days post-MCT injection. In contrast, when GLY was administered, thereby inhibiting HMGB1, the survival rate improved to 90% (18 of 20 animals) in the MCT + GLY group (Kaplan-Meier analysis; p = 0.0027) (Figure 6A). In a complementary approach, we also measured the degree of pulmonary inflammation by micro-PET imaging at 28 days post-MCT injection. In the micro-PET technique, radiotracer uptake is proportional to the degree of inflammation present. Increased levels of radiotracer uptake were observed in lung tissue from animals in the MCT group when compared with the control group, and this increased uptake was attenuated with GLY treatment (Figure 6B). To quantify radiotracer uptake, we calculated the SUV, which expresses the ^18^ F-FDG uptake per unit volume in the lung field. SUVs increased in the MCT group compared with the control group. Upon treatment with GLY, however, SUVs decreased significantly, from 0.4724 ± 0.098 to 0.2931 ± 0.016 (p = 0.018) in the MCT + GLY group (Figure 6C).

### HMGB1 induced ET-1 release from HPAECs and proliferation of HPASMCs

**Figure 7:**
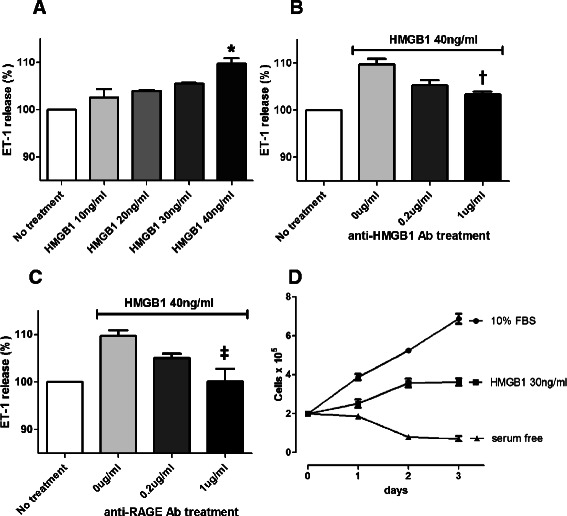
**HMGB1 induced ET-1 release from HPAECs and proliferation of HPASMCs. (A)** HMGB1-stimulated ET-1 release from HPAECs. Release of ET-1 was approximately 10% higher in the HMGB1-treated (40 ng/ml) group than in the control group (*p <0.001). **(B, C)** ET-1 release from HPAECs was attenuated by treatment antibodies against HMGB1 or RAGE in a dose-dependent manner (†p = 0.011; ‡p = 0.006). **(D)** HPASMCs were cultured with serum-free medium alone or 10% FBS, or in the presence of 30 ng/ml HMGB1. HMGB1 induced proliferation of HPASMCs (p <0.001). Data are presented as means ± SEM.

To determine whether HMGB1 plays a role in endothelial hyperactivity in pulmonary hypertension, we quantified ET-1 release from cultured HPAECs after stimulation with HMGB1. Release of ET-1 was higher in the HMGB1-treated (40 ng/ml) group (109.7%; 381.92 ± 7.1 pg/ml) than in the non-treated control group (348.23 ± 12.2 pg/ml; p <0.001) (Figure 7A). Furthermore, HMGB1 increased the release of ET-1 from HPAECs in a dose-dependent manner (range, 10–40 ng/ml). Moreover, the addition of antibodies against HMGB1 (1 μg/ml) significantly attenuated HMGB1-induced ET-1 release (at 40 ng/ml HMGB1) from 384.16 ± 1.3 pg/ml to 359.63 ± 5.1 pg/ml (p = 0.011) (Figure 7B). Likewise, anti-RAGE antibodies (1 μg/ml) also reduced the amount of HMGB1-induced ET-1 release from 381.56 ± 17.3 pg/ml to 348.51 ± 16.3 pg/ml (p = 0.006) (Figure 7C).

Migration and proliferation of vascular smooth muscle cells are also major pathological features in pulmonary hypertension vascular lesions. We confirmed the previously reported proliferative effect of HMGB1 on human pulmonary artery smooth muscle cells. HPASMCs were cultured with serum-free medium alone or 10% FBS, or in the presence of 30 ng/ml HMGB1. Treatment of 30 ng/ml HMGB1 induced proliferation of HPASMCs compared to serum-free medium alone (p <0.001) (Figure 7D).

## Discussion

In this study, we found that HMGB1 levels in lung tissue and serum significantly increased in MCT-induced pulmonary hypertension rats. We also demonstrated that inhibition of HMGB1 by GLY treatment improved the survival rates of MCT-induced pulmonary hypertension rats by reducing pulmonary hypertension and attenuating pulmonary vascular remodeling and RV hypertrophy. In addition, *in vitro* study data supplied in Figure 7 presents the potential effects of HMGB1 on endothelium hyperactivity and pulmonary vascular remodeling. These results suggest that HMGB1 may play a significant role in the pathophysiology of pulmonary hypertension.

MCT is a toxic pyrrolizidine alkaloid. A single subcutaneous injection of MCT induces severe pulmonary hypertension in rats after 4 weeks [26]. The mechanisms by which MCT causes pulmonary hypertension are not fully resolved; however, the proposed mechanism of action is as follows. MCT is activated to the reactive compound MCT pyrrole in the liver. This MCT pyrrole causes endothelial injury in the pulmonary vessels and subsequently induces remodeling of the precapillary vessels in ways such as medial thickening and *de novo* muscularization [27]. In the MCT-injected rats, the acute inflammatory response is turned off within a week after MCT injection, and vascular remodeling is observed after 1–2 weeks [23,26]. Our results are consistent with these data, as serum levels of HMGB1 in pulmonary hypertension rats were normal in the acute inflammatory period (comprising the first week after MCT injection), then increased and remained elevated throughout the vascular remodeling period (2 weeks after MCT injection and onwards). These findings suggest that the increased levels of HMGB1 might be more closely related to pulmonary vascular remodeling of pulmonary arteries than acute MCT-induced pulmonary vascular inflammation in the MCT-induced pulmonary hypertension rats.

In our experiments using the MCT-induced pulmonary hypertension rats, RVSP and RV hypertrophy significantly decreased in the GLY-treated group compared to the non-treated group. In addition, GLY treatment significantly reduced the medial wall thickness of pulmonary arteries and the muscularization of small pulmonary vessels. These results indicate that inhibition of HMGB1 by GLY treatment can lead to hemodynamic improvement and attenuation of pulmonary vascular remodeling, at least in the MCT-induced pulmonary hypertension rats. Furthermore, GLY treatment also conferred a survival benefit in the MCT-induced pulmonary hypertension rats. A recent study from Bauer and co-workers reported that serum HMGB1 levels were higher in idiopathic pulmonary hypertension patients than in healthy controls, and HMGB1 may contribute to the pathogenesis of experimental pulmonary hypertension induced by chronic hypoxia [20]. This study shared the same conclusion with that of our study: inflammatory cytokine HMGB1 likely plays a significant role in the pathophysiology of pulmonary hypertension; however, used animal model of pulmonary hypertension was different with that of our study. The MCT-induced pulmonary hypertension rat is one of the most commonly utilized animal models of pulmonary hypertension, although there are many additional pulmonary hypertension animal models employing various chemical, physiological, and molecular interventions. Each pulmonary hypertension animal model has its own unique limitations in representing human pulmonary hypertension; there is no perfect animal model that faithfully reproduces all pathophysiological features of human pulmonary hypertension [27]. Although our data support a meaningful role of HMGB1 and demonstrate the therapeutic effects of GLY in MCT-induced pulmonary hypertension rats, further investigation into HMGB1 using additional pulmonary hypertension animal models and clinical studies of patients diagnosed with pulmonary hypertension are required. Thus, the study by Bauer and co-workers was helpful in clarifying our results about the role of HMGB1 in pulmonary hypertension.

Vasoconstriction promoted by endothelial dysfunction is one of the major pathologic features of pulmonary hypertension. ET-1 is a peptide secreted by vascular endothelial cells that mediates vasoconstriction of pulmonary arteries and is targeted by one of the classes of current therapeutics for pulmonary hypertension. In our *in vitro* study, ET-1 release by HPAECs was significantly increased by HMGB1 treatment in a dose-dependent manner. Moreover, this HMGB1-stimulated increase in ET-1 release could be inhibited with antibodies against either HMGB1 or RAGE. These data demonstrate that HMGB1 can induce ET-1 release from HPAECs via a RAGE-mediated mechanism. RAGE is a multi-ligand receptor and is expressed in many types of cells, including macrophages, neural cells, smooth muscle cells, and endothelial cells. Furthermore, it is well known that HMGB1 can activate endothelial cells by interacting with RAGE, which ultimately leads to NF-κB activation [18,19,28]. Several studies have also reported that RAGE-dependent signaling pathways regulate ET-1 release from endothelial cells. Advanced glycation end product, another ligand of RAGE, can induce mRNA transcription of ET-1 by cultured endothelial cells in an NF-κB–dependent manner [29]. In support of a role for RAGE in this process, increased ET-1 expression in cultured endothelial cells is attenuated by RAGE antagonists [30], and serum levels of ET-1 decrease in RAGE knockout mice [31]. Hypersecretion of ET-1 is an important factor in pulmonary hypertension pathogenesis, and ET-1 antagonism has been shown to have clinical use in the treatment of pulmonary hypertension [32]. In addition, the HMGB1-RAGE interaction has also been shown to induce migration and proliferation of vascular smooth muscle cells and mesoangioblasts [24,33]. The response of smooth muscle cells to HMGB1 has been well-characterized in atherosclerotic plaques [16,17]; however, migration and proliferation of vascular smooth muscle cells are also major pathological features in pulmonary-hypertension vascular lesions. We confirmed the previously reported proliferative effect of HMGB1 on human pulmonary artery smooth muscle cells in Figure 7D. Considering its potential effects on endothelium hyperactivity and pulmonary vascular remodeling, it is reasonable to envision a valuable role for HMGB1 in the pathophysiology of pulmonary hypertension.

Current therapeutic strategies for pulmonary hypertension have mainly focused on vascular hyperactivity. This approach improves many of the symptoms and hemodynamic properties of pulmonary hypertension; however, it has a limited ability to improve vascular remodeling and patient survival. Inflammatory processes in pulmonary hypertension are important pathogenic features for identifying new therapeutic strategies [5-8]. Considering the cytokine activities of HMGB1, it is reasonable to propose that the effects of GLY on MCT-induced pulmonary hypertension shown in this work are related to the inflammatory processes of pulmonary hypertension. In line with this hypothesis, our data showed that GLY treatment reduced the degree of pulmonary inflammation, as measured by micro-PET, when compared with the non-treated MCT group.

We employed GLY, a molecule that binds directly to HMGB1 and inhibits its cytokine activities, as a chemical inhibitor of HMGB1 in this study [34,35]. GLY is a natural compound produced by the licorice plant that has been used clinically to treat patients with chronic hepatitis for more than 30 years, especially in Japan [36,37]. In the previous study, Sitia and co-workers showed that as an HMGB1 inhibitor, GLY is as effective as Box-A, a representative HMGB1 direct inhibitor [34]. Our investigation of the therapeutic effects of GLY on pulmonary hypertension has merit, given that the pharmacological properties and safety of GLY, two of the most important hurdles for any new therapy to overcome, are already well-established.

However, using GLY as an HMGB1 inhibitor also has several limitations. GLY has various pharmacological effects [36]; therefore, we cannot jump to the conclusion that the protective effect of GLY is only due to the inhibition of HMGB1 in spite of previous proof about its direct binding activity to HMGB1. Another controversial aspect of our study is that it is possible for GLY to induce an effect that is contradictory to our results. A single study from Ruszymah and co-workers showed that GLY caused an increase in right atrial pressure as well as thickening of the pulmonary vessels [38]. However, data from the same study also showed a significant increase of tail blood pressure in GLY-treated rats, and right atrial pressure was strongly correlated with tail blood pressure. Pseudoaldosteronism, with sodium retention, hypokalemia, and hypertension, is a well-known side-effect of GLY [36]. Increased RA pressure and thickening of pulmonary vessels might be indirect results from systemic hypertension, not from a direct pharmacologic effect of GLY. Aharinejad and co-workers showed that spontaneously hypertensive rats developed pulmonary hypertension and muscularization of pulmonary vessels [39]. It is also worth noting that Ruszymah and co-workers applied a very high dose (1.0 mg/mL, 40–50 mL/day, 160–200 mg/kg/day for rats with a body weight 250 g) and a very long term treatment (12 weeks) of GLY compared with our experiments (50 mg/kg/day, 4 or 6 weeks). We showed that GLY given alone without MCT in our experimental condition did not lead to any differences in RVSP, RV hypertrophy, medial wall thickness, or pulmonary vascular muscularization when compared with normal control rats.

Due to the above limitations in our study, further studies with additional HMGB1 inhibitors will be important for providing additional evidence to support the data presented here. Two other options for inhibiting HMGB1 involve treatment with either anti-HMGB1 antibodies or recombinant Box-A, an antagonist that competes with extracellular HMGB1 [12].

Our animal study protocol is also a limitation due to our decision to start GLY treatment immediately after MCT administration. In retrospect, our choice of a preventive protocol was unwise. Pulmonary hypertension is frequently misdiagnosed and has often progressed to a late stage by the time it is accurately diagnosed. A better choice would be a curative protocol starting GLY treatment at day 7 and continuing until day 28.

## Conclusions

In conclusion, we demonstrated that the proinflammatory cytokine HMGB1 could contribute to the pathophysiology of pulmonary hypertension. Treatment of pulmonary hypertension with GLY, an HMGB1 inhibitor, has the potential to attenuate pulmonary vascular remodeling and disease progression of pulmonary hypertension. This approach might constitute a promising novel therapeutic strategy for human patients with pulmonary hypertension.

## Abbreviations

α-SMA: alpha smooth muscle actin
ET-1: endothelin 1
GLY: glycyrrhizin
HMGB1: high mobility group box-1
HPAEC: human pulmonary artery endothelial cells
HPASMC: human pulmonary artery smooth muscle cells
LV: left ventricle
MCT: monocrotaline sodium
PET: positron emission tomography
RV: right ventricle
RVSP: right ventricular systolic pressure
S: septum
SUV: standardized uptake value
vWF: von Willebrand factor
