# Erratum to: Glycyrrhizin, inhibitor of high mobility group box-1, attenuates monocrotaline-induced pulmonary hypertension and vascular remodeling in rats

**DOI:** 10.1186/s12931-016-0458-9

**Published:** 2016-11-04

**Authors:** Pil-Sung Yang, Dae-Hoon Kim, Yong Joon Lee, Sang-Eun Lee, Won Jun Kang, Hyuk-Jae Chang, Jeon-Soo Shin

**Affiliations:** 1 Division of Cardiology, Severance Cardiovascular Hospital, Yonsei University Health System, 50 Yonsei-ro Seodaemun-gu, Seoul, 120-752 Republic of Korea; 2 Departments of Microbiology, Yonsei University College of Medicine, 50 Yonsei-ro Seodaemun-gu, Seoul, 120-752 Republic of Korea; 3 Departments of Nuclear Medicine, Yonsei University College of Medicine, 50 Yonsei-ro Seodaemun-gu, Seoul, 120-752 Republic of Korea; 4 Severance Biomedical Science Institute, Yonsei University Health System, 50 Yonsei-ro Seodaemun-gu, Seoul, 120-752 Republic of Korea

## Erratum

In the original article [1], the following information was omitted from the funding section:

“This study was supported by a faculty research grant of Yonsei University College of Medicine for 2009 (6-2009-0101).”
